# Defining the Characteristics of Successful Biosecurity Scent Detection Dogs

**DOI:** 10.3390/ani13030504

**Published:** 2023-01-31

**Authors:** Ariella Y. Moser, Wendy Y. Brown, Pauleen Bennett, Peta S. Taylor, Bethany Wilson, Paul McGreevy

**Affiliations:** 1 School of Environmental and Rural Science, University of New England, Armidale, NSW 2350, Australia; 2 Anthrozoology Research Group, Department of Psychology, Counselling & Therapy, La Trobe University, Melbourne, VIC 3083, Australia; 3 School of Life and Environmental Science, Faculty of Science, University of Sydney, Camperdown, NSW 2006, Australia; 4 One Welfare Research Institute, Faculty of Science, Agriculture, Business and Law, University of New England, Armidale, NSW 2350, Australia

**Keywords:** detector dog, sniffer dog, working dog, selection, personality, traits, behaviours, focus group, survey, drive

## Abstract

**Simple Summary:**

Dogs should display certain physical, behavioural, and cognitive characteristics to be suitable for training and deployment in a scent detection role. Identifying these characteristics is a vital first step to determine whether particular dogs will be suitable for selection and training. This study identified the behavioural traits that stakeholders consider relevant for dogs trained in biosecurity detection, and then assessed these traits in an active cohort of Australian biosecurity detector dogs. Our research revealed seven relevant traits: search motivation, emotional stability, search arousal, food motivation, play motivation, search independence, and search focus. Current biosecurity detector dogs had consistently high ratings from their handlers for search motivation, emotional stability, and food motivation, whereas other traits showed more variation. We found that dogs rated by handlers as high in search arousal and search motivation were more likely to also be rated highly for their overall detection performance. These findings will help to inform decisions about the selection and training of scent detection dogs.

**Abstract:**

To perform their role effectively, scent detection dogs require certain characteristics. Identifying these characteristics will inform the selection of prospective dogs and preferred approaches to their training. The current study drew upon the perspectives of industry stakeholders to identify the behavioural traits considered relevant for detection dogs in biosecurity screening roles. Dog handlers, trainers, and supervisors (*n* = 25) in Australian biosecurity operations participated in focus group interviews to determine the perceived characteristics that, in their experience, influence detection performance. Their descriptions were used to create a questionnaire which was then administered to handlers to assess the working behaviours of current biosecurity dogs. Responses were collected for 88% of the operational dogs (*n* = 36). An exploratory factor analysis revealed seven tentative dimensions: search motivation, emotional stability, search arousal, food motivation, play motivation, search independence, and search focus. Search motivation and search arousal were both positively associated with handler ratings of detection performance (*p* ≤ 0.006). In general, biosecurity dogs were scored consistently high in ratings of search motivation, emotional stability, and food motivation. Our approach has advanced our understanding of the working behaviours and characteristic profile of biosecurity detector dogs and will be used to inform candidate selection processes.

## 1. Introduction

Scent detection dogs are widely recognised as highly effective in screening and locating elusive targets across a number of fields. Their use in policing, rescue, and military [1,2] is well-established, but more recent applications have emerged in biosecurity, human health screening, and wildlife conservation [3,4,5]. 

Several behavioural and cognitive processes converge when a trained scent detection dog searches an area and signals the source of a target odour [6]. They must exhibit scenting behaviours appropriate to the task, disregard distractions, retain in their working memory the task and target odour, and evaluate odours against their trained prototype odours to discriminate or generalise accordingly. Meanwhile, they should often also be responsive to handler cues and directional input. Some dogs are better suited to this complex task than others and, as a reflection of their individual characteristics, their performance can vary accordingly [6,7]. As such, selecting dogs on the basis of appropriate characteristics can improve outcomes and optimise the performance of scent detection dogs [8,9]. Good selection processes reduce costs incurred for training and housing unsuitable dogs, and also potentially improve dog welfare by only recruiting dogs that will be behaviourally and cognitively equipped for the demands of the role. However, identifying these specific characteristics is not always straightforward due to the inherent complexity of defining and predicting animal behaviour.

The characteristics of successful scent detection dogs have been investigated with a variety of methods, including subjective ratings and direct behavioural assessments [10]. Methods that draw upon the perspectives and practical experiences of industry professionals can provide real-world insight into the tendencies and challenges that professionals experience in a given role [9,11]. Additionally, this approach can help to bridge gaps between industry needs and empirical research, improving the applicability of research outcomes [9,11]. Meanwhile, objective behavioural assessment allows handlers’ subjective expectations to be tested. Together, both approaches underpin a comprehensive understanding of dogs’ characteristics and working performance in scent detection roles. 

To date, research into the selection of scent detection dogs has revealed an industry preference for dogs with a strong intrinsic motivation to search, high levels of playfulness, boldness, and environmental stability [6,7,11,12]. Furthermore, behavioural research has revealed associations between various constructs and performance outcomes. These include positive associations among performance and search desire and ability [2,12], activity [13], trainability [2], environmental stability [2,14], inhibitory control [15], short-term memory [8,16], and search thoroughness [17]. These traits seem to underpin many specific working behaviours. 

This body of literature offers a robust basis to predict desirable traits in scent detection dogs, but some important knowledge gaps remain. Importantly, there are discrepancies in the way certain characteristics are labelled, defined, or measured, which may help to explain apparently contradictory findings in the literature. For example, the association of inhibitory control with detection performance has been reported to be positive [15], negative [18], and inconsistent [16]. Additionally, the overall literature is still somewhat limited in scope. Certain applications, such as the detection of explosives and narcotics, are predominantly represented [10]. Although relevant, such findings may not be directly applicable to all scent detection roles, which present different environmental challenges, working conditions, and target odours. Furthermore, the most commonly used performance measure is whether or not a dog completes training (i.e., reaches qualification or certification) [2,12,14,19,20], but this outcome does not necessarily predict the quality of ongoing performance in an operational context. These gaps necessitate further research using various methods to investigate, validate, and confirm findings.

The current research is a first step in determining the canine behaviours and characteristics that contribute to scent detection dog success within Australian biosecurity detector dog (BDD) operations. Australian BDDs are used to detect organic material that may pose a biological threat to native species or domestic agricultural industries. To do so, they are trained using positive reinforcement to respond to more than 200 different commodities. They predominantly work for extended periods in busy, relatively target-rich environments, such as airports, mail centres, and wharves, performing fairly repetitive search tasks. Additionally, any BDD is expected to be able to be handled by any handler in the agency. Some of these tasks and conditions are comparable with those of other scent detection dogs, such as those used for explosives detection. We therefore expected overlap of desirable traits for BDDs and other types of scent detection dogs. On the other hand, some of these conditions are less common among scent detection dogs and may involve specific attributes. For example, working for extended periods with frequent detections may be aided by a particularly insatiable motivation for rewards. Working for any handler may require a very independent approach to detection and relatively low attachment to individual handlers. 

In this initial study, we sought to determine stakeholders’ perspectives on the behavioural characteristics and performance of their current cohort of BDDs. This approach will inform ongoing research to design a standardised behavioural test battery for the evaluation and selection of candidate dogs for this agency. Specifically, we sought to probe the relevant agency staff’s perspectives of canine working behaviours and their associations with performance within the agency. This was an important first step to strengthen the relevance of the ongoing research by drawing upon the authentic experiences of stakeholders and dogs in this role. Furthermore, since definitions for canine traits are rarely unequivocal, this approach sought to determine a shared vocabulary of traits with behavioural descriptors in order to collect data about individual dogs’ working behaviours and performance within this agency.

To serve this purpose, we aimed to (1) develop a framework that captures scent detection traits relevant to BDDs; (2) using this framework, ascertain the behavioural profile of currently operational BDDs; and (3) assess the utility of a questionnaire for investigating work-related behaviours for BDDs. The research was conducted in two parts. Firstly, Australian government BDD stakeholders were interviewed in focus groups. Subsequently, a questionnaire based on findings from the focus group discussions was administered to the agency’s dog handlers to assess the working characteristics of their currently operational dogs. 

## 2. Focus Group Materials and Methods

### 2.1. Recruitment, Participants, and Procedures

All eligible employees (*n* = 75) were invited via email to participate on a voluntary basis. An information sheet that provided a brief overview of the study was distributed at this time. A total of 8 mini focus group interviews, with a mean of 3 participants per group (min = 2; max = 5), were conducted with employees (*n* = 25) of the Australian Government Department of Agriculture, Water, and Environment (DAWE) whose roles were directly related to BDD operations. They were grouped according to their roles to facilitate open discussion without potential impediment from the presence of line managers. Small group sizes with frequent time availabilities sought to encourage participation based on availability and allow individuals to speak at length. Staff participated during their usual working hours and each interview lasted approximately 60–90 min. The participants included dog handlers and kennel staff (*n* = 16), dog trainers (*n* = 3), supervisors (*n* = 3), and management (*n* = 3). Encouraging the participation of staff in various roles sought to invite different perspectives about various facets of BDD behaviour that might otherwise be overlooked. 

The focus groups were conducted over Zoom and moderated by two members of the research team. Each participant joined the teleconference meeting from a separate device at a work location and each voluntarily consented to their participation. Participants were reminded that sharing personal opinions or experiences was encouraged. 

A structured guide was used for the discussions, and the following three questions were asked in each group discussion:

1. What qualities do you think are important for success in a biosecurity detector dog?
2. What characteristics do you think make some dogs less successful?
3. If you were given the task of selecting dogs for this organisation, what characteristics would you prioritise in new candidates?

Finally, participants were asked to reiterate an important point they thought had been made in the discussion or one that had not yet been mentioned. These questions and structure were chosen to encourage participants to respond to the central question—what qualities influence the performance of BDDs?—from different perspectives. 

The moderators asked additional questions throughout to clarify and further explore participants’ views. These questions sought to encourage participants to describe specific behaviours, rather than traits or characteristic adjectives. Examples of such prompts included “what does that look like to you?” and “what do you mean by [statement]?” 

### 2.2. Data Coding and Analysis

Focus group interviews were audio recorded, with the formal consent of all participants. Transcription and coding were conducted using NVivo (Version 12, 2018). The answers to the three questions were analysed together. The transcriptions were coded, and the coding rubric was refined iteratively using grounded theory analysis [21]. Firstly, transcripts were open coded using labels drawn from participants’ descriptions. Following this, axial coding was used to draw connections between these nodes and group them into categories drawn from participants’ explanations of the behaviours. Finally, these categories were selectively coded to arrive at the overall themes of the responses.

### 2.3. Ethics Statement

Approval to conduct this research was granted by the University of New England Human Research Ethics Committee (HE21–255).

## 3. Focus Group Results

Overall, participants described successful BDDs as those which were willing and capable of performing the role. Dogs’ willingness was reflected in their perceived motivation and independence, which were qualities mentioned most frequently in discussions. Meanwhile, their capability was reported to depend upon their emotional stability, cognitive ability, and physical suitability. 

### 3.1. Motivation

Dogs’ willingness to work was the theme most frequently discussed by participants. This was generally described as “drive”, a term commonly used in industry that refers to different aspects of canine motivation. In the context of detection work, participants referred to reward drive (food, play/prey, toy/dummy drives) and hunt drive. 

**Figure 1:**
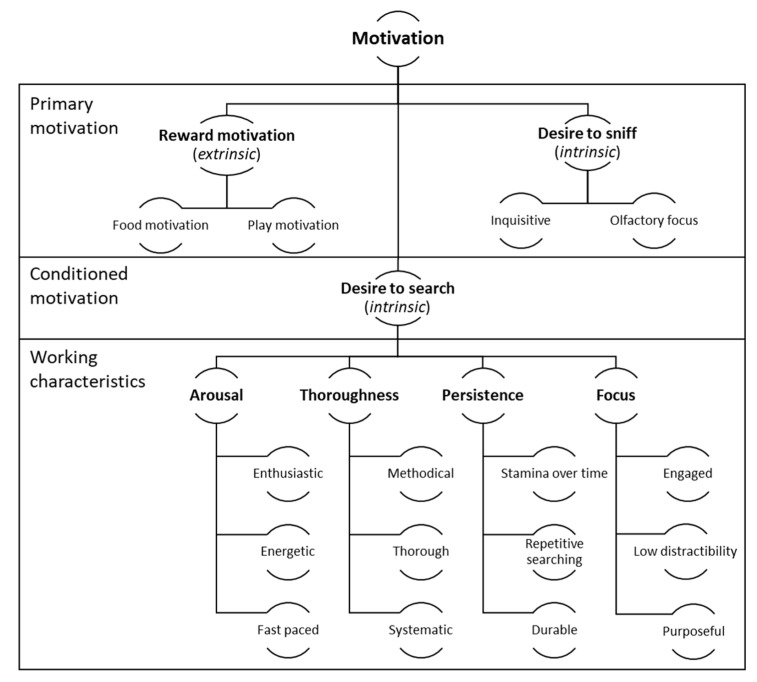
Characteristics and descriptors associated with work motivation among biosecurity detector dogs, as described by focus group participants.

In every group, participants described a dog with high drive as most suited for detection work. They were asked to expand upon this in each instance, and the resulting behavioural descriptors were used in the analysis. Reward drives were described as dogs’ motivation or desire to access a primary reinforcer. Hunt drive tended to refer to a more complex set of behaviours that, taken as a whole, indicated a dog with a strong intrinsic desire to search using odour. Figure 1 presents participants’ descriptions of these traits in terms of the dogs’ primary motivators, their conditioned motivation to search for target odours, and the behavioural indicators of high motivation in a deployment context. These are discussed in the following sections.

#### 3.1.1. Reward Motivation

Most participants (18/25) said that BDDs need to have a strong desire for a given reward. All eight groups raised this and no participants disagreed. Indeed, some described this level of desire as an “obsession”. The behavioural indicators of this attribute were described as a consistent eagerness to eat (for food rewards) or to grab or chase an item (for play rewards).

*“The higher the drive for those foods or play reward, I think the more successful you’ll be with your training because if the dog values the reward, then they’re more likely to put the effort into the actual task that we’re asking them to do, especially when we’re doing large volume screening with quite a low target rate.”*

(Speaker 11)

Participants indicated that this motivation should persist over time and/or multiple reinforcing events.

*“A dog that doesn’t reach satiation levels, so a dog that will constantly want more rewards… it constantly wants more food, more play, whatever the reward is. Otherwise they get to that level where they’re content and have no interest in continuing to work.”*

(Speaker 12)

A dog’s desire for rewards was considered an important factor for success as a scent detection dog. It was proposed that this motivation can be increased through training if required, but it was preferable that the dog naturally had a strong motivation for a reward. At the time the interviews were conducted, a desire for both food and dummy play rewards were perceived to be important for dogs to be effectively deployed in different locations where different types of rewards were used.

#### 3.1.2. Desire to Sniff

Dogs’ innate desire to seek and investigate odours was discussed in five of the eight groups, by 11 of the 25 participants. These participants described this as an underlying trait for a strong hunt drive or motivation to search. Several participants described dogs with this quality as curious or inquisitive, and most suggested that this is reflected in their tendency to explore their environment, especially through olfaction.

*“They’re nose to the ground straight away, they’re looking for something… actively engaging with things in their environment…. and investigating smells... So, for me, that’s what the hunt drive looks like initially.”*

(Speaker 23)

#### 3.1.3. Search Motivation

Finally, most participants in all eight groups (18/25), with no disagreement, stated that the dogs’ desire to search for their trained target odours was one of the most important qualities for success. They explained that this quality is underpinned by the dogs’ desire for a reward and their innate desire to sniff, resulting in the working task being highly reinforcing for the dogs.

*“We don’t want to make them work. We want them to love doing it. So, you want it to be just in their nature—they love it. Work should be the funnest [sic] thing ever.”*

(Speaker 8)

*“Hunting and finding what they’re after is almost like its own reward and they’re keen to get back into it.”*

(Speaker 11)

The observed behaviours of dogs with this quality were described in several ways, as outlined in Figure 1.

According to participant descriptions, one behavioural indicator of dogs’ motivation to search is their arousal level, as reflected in a dog that shows excited or energetic behaviours.

*“They’re showing that intensity.”*

(Speaker 10)

*“A high drive dog that really wants to get out there, work at a fast pace… go in, work hard, work fast”*

(Speaker 17)

*“A dog that has good energy and responds well to the handlers’ energy as well, you know, eager to come out.”*

(Speaker 22)

However, importantly, participants clarified that over-arousal was not desirable, and could lead to tiring out quickly or an inability to focus, sometimes resulting in missed targets.

*“When we talk about dogs that are highly motivated, often that gets confused with highly aroused… [but] dogs with high levels of arousal [are] generally pretty poor searchers. The search suffers as a result; it’s not methodical, it’s not planned, it’s not detailed and… it’s not sustainable over an extended period of time. So those dogs, regardless of how fit they are, generally have much shorter effective deployment times than a dog that has a lower state of arousal with a similar level of motivation.”*

(Speaker 24)

Dogs with a strong desire to search were also described as being focused and purposeful in their searching behaviour. This may manifest in their searching with more effort.

*“Coming out with a purpose to work… [they] work with intent”*

(Speaker 17)

*“[Dogs with low motivation] go through the motions, but it takes a lot of energy and work from the handlers to keep them on track and to keep them actively smelling… they might be walking where you need them to [but] they’re not actively doing their job”*

(Speaker 5)

It was also stated that this motivation should be persistent. If dogs are highly motivated, they should persist even when the search is difficult and should maintain motivation despite long durations, changing search conditions, or varying reinforcement schedules.

*“[The] natural willingness to engage and hunt for it and maintain that hunt for a period of time rather than disengaging and losing interest.”*

(Speaker 22)

Thoroughness in their search was also proposed to characterise dogs that are highly motivated to find a target.

*“There’s quite often an emphasis on speed with the detection dogs, but I think that there is a lot of value in those dogs that are really methodical in the way that they search, whether it be luggage or parcels on a mail belt. I find that impressive when you see a dog that basically doesn’t want to let anything go unsniffed [sic].”*

(Speaker 11)

Overall, if dogs are highly motivated and are otherwise behaviourally, cognitively, and physically capable of the work, they would be expected to demonstrate arousal, focus, persistence, and thoroughness while searching.

### 3.2. Emotional Stability

**Figure 2:**
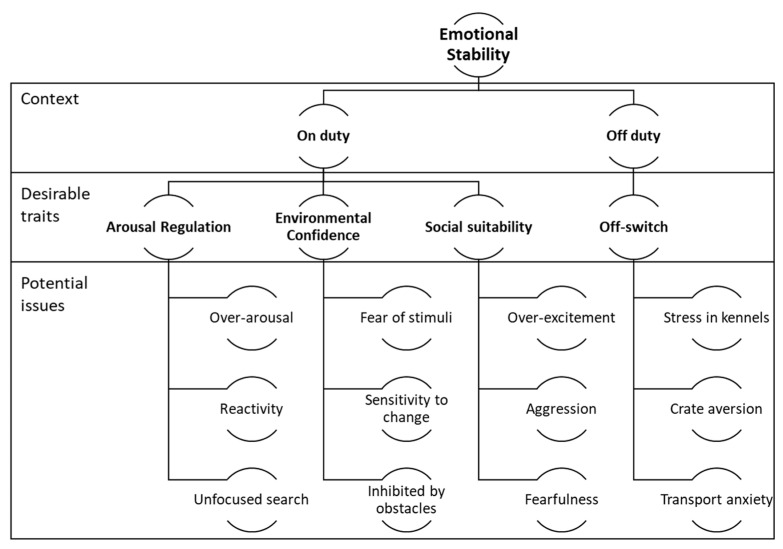
Emotional stability traits and descriptors in biosecurity detector dogs as described by focus group participants.

BDDs in this agency are exposed to many potential stressors throughout their working life. Such stressors include busy and noisy work environments, frequent crating and transport, change of handlers, and kennel environments shared with other dogs. To be capable of working in these conditions, participants said that dogs should be free from excessive nervousness or anxiety while working and off duty (see Figure 2). Poor emotional stability and inability to cope with the stressors of the working role were identified as the most common causes of early retirement for the agency’s scent detection dogs.

#### 3.2.1. On Duty

Appropriate arousal, in terms of excitement and stress responses, was discussed in all eight groups. Arousal can be an indicator of a dog’s willingness to work and dogs typically tend to be selected on the basis of showing high arousal behaviours, such as excitement and energy, in a search context. However, several participants also posited that over-arousal could contribute to chronic and acute stress responses, inappropriate reactivity to stimuli, slow recovery, and poor-quality searching.

According to participant responses, some dogs are less capable of coping with the stressors inherent in their working role. These dogs were often described as anxious or “high-strung”. Participants commented that arousal behaviours stemming from anxiety, such as panting, tail-wagging, and restlessness, could be misinterpreted as excitement or drive.

*“I think for some of our nervous dogs that that’s been confused as being drive. So, they might look drivey [sic], but actually they’re nervous and they’re anxious.”*

(Speaker 16)

It was expressed in discussions that this anxiety and reactivity tended to detract from dogs’ detection performance.

*“You only get 50 percent as opposed to 95 percent of what the dog is capable of… A lot of dogs will look like they’re actually going through the motions… but they’re really not engaged in the whole game and 100 percent committed to what they’re doing because their mind is half full about stuff that’s happening around them.”*

(Speaker 1)

Similarly, dogs that work in a high state of arousal with little behavioural regulation may tend towards undesirable reactivity behaviours, including fixating on or barking at stimuli such as people or other dogs. This form of distractibility was identified as an issue for some dogs.

*“We have had one or two dogs that I can recall that once they even see another dog walking even 50 meters away, they become fixated on that and that detracts from the focus of their work.”*

(Speaker 20)

In terms of social suitability, dogs are expected to behave neutrally towards strangers and other dogs while being relaxed and happy to be approached and handled. Participants agreed that, when working, dogs should not show emotional responses such as fearfulness, aggression, or over-excitability.

*“[They have been] socialised with people. Not that they’re wanting to run up and say hello, but that they understand that [for example] kids are there and they don’t mind. They’re not showing any aggression or fearfulness around different types of people.”*

(Speaker 12)

Similarly, participants expressed that dogs should not be fearful or stressed in response to environmental stimuli but instead should be able to confidently perform their working task regardless of the environmental conditions or obstacles.

*“[Successful dogs are] able to work through pressure, they’re not sensitive to changes in the environment. They’re observant and acknowledge changes instead of being fearful of things that change and a different environment.”*

(Speaker 1)

The term “resilience” was mentioned in six of eight groups, with reference to being impervious to environmental stressors, sub-optimal handling, or changes in routine. This trait also embraced the dogs’ ability to recover or cope with potentially stressful events and reflects an overarching quality of emotional regulation.

#### 3.2.2. Off Duty

Participants agreed that it was important that dogs show appropriate emotional stability when off-duty, particularly in being able to rest and adapt to changes in routine. In the course of deployment, dogs are regularly crated to rest in between tasks and are transported to different areas throughout the day. Some can “switch off” and rest during these times, whereas other dogs show significant stress behaviours such as panting when confined.

*“[A retired dog] was extremely anxious in the vehicle… and actually got so bad that she would then not be able to be used for deployment because she was panting too much.”*

(Speaker 13)

Similarly, participants discussed chronic anxious behaviours at kennels. This not only represents a risk to the dog’s welfare but may also detract from their working performance.

*“Because they spend so much time at kennels, we have to have dogs that are able to have an off switch and able to turn off, chill out. We don’t want dogs that are here for the time that they’re not working to be anxious, pacing, barking, fence fighting [and showing] aggression to other dogs.” *

(Speaker 9)

Overall, dogs with poor emotional stability would be expected to show poor performance and be predisposed to negative welfare outcomes in the long term.

### 3.3. Independence

Dogs’ independence and self-assuredness in their working role were discussed in seven of the eight groups and were identified as important for success in a detection role. Participants expressed that successful scent detection dogs tend to be only moderately obedient. An appropriate balance between obedience and independence allows a dog to respond to the handler, while being primarily interested in interacting with the environment rather than with people.

*“Dogs that ended up being good dogs, dogs with drive, those dogs are hard to get back. Dogs that aren’t that interested in a handler… they’re more engaged with the environment.”*

(Speaker 23)

Ideally, while working, preferred dogs were said to be minimally aware of the handler. Instead, the dogs are expected to make independent decisions about following or responding to an odour. Dogs lacking this quality were described as handler-dependent.

*“[It is] an issue if they become handler dependent and they’re just looking at the handler for the cues to find the target rather than actually just finding it themselves.”*

(Speaker 11)

In the agency, dogs are handled by several different handlers and, once fully trained, are expected to work with any handler. As such, they should work consistently regardless of the handler. This was described as being a particular challenge for some dogs.

*“They’re so dependent on my input and me being there and developing that bond is so much more important to them before they were willing to work.”*

(Speaker 7)

The extent to which dogs will search independently is likely impacted by their level of motivation. However, discussions revealed that even a highly motivated dog should also have a high level of self-assuredness and confidence to search independently. According to examples, dogs lacking this quality could become overly sensitive to handlers’ behaviour while searching, thereby detracting from their detection performance.

### 3.4. Cognitive Ability

Participants in half of the groups also mentioned characteristics related to dogs’ intelligence or cognitive aptitude. Some suggested that the dogs should be “trainable”. Although this depends at least partly on the dogs’ motivation to learn, participants expressed that some dogs appeared to be less capable of learning new tasks, despite their apparent motivation. Furthermore, one participant identified that some dogs were better than others at generalising between odours, which is an important part of the training process.

Additionally, four participants reported a preference for dogs that were good at problem solving, and notably at attempting new behaviours when trying to access a reward or reach a target odour. On the other hand, at least one handler mentioned that more intelligent dogs tended to seek potential shortcuts, such as handler cues in the training environment or environmental cues. That said, a trainer identified that this apparent reliance on handler cues could be overcome with training.

*“[A trainable dog] looks like a dog that’s willing to learn and is able to problem solve. There [are] some dogs that you can teach the basics to, and they’re really good at "sitting" but… have difficulty… working more independently and problem-solving different situations.” *

(Speaker 2)

This theme was not frequently identified in discussions with handlers but was discussed in the trainer and technical supervisor groups.

### 3.5. Physical Capability

Finally, many of the participants mentioned or agreed (in six of eight groups) with the requirement for dogs to be physically capable of performing the role. This included their health and physical wellness, good physical condition and fitness, a structural conformation appropriate for efficient and uninhibited movement, and a physical size and type conducive to navigating obstacles.

This tended to be mentioned as a basic requirement that should be considered at the time of breeding and initial assessment. The discussions focused on behavioural attributes as the most important characteristics determining success among dogs that were physically suited for the task.

## 4. Survey Materials and Methods

### 4.1. Survey Development

Subsequent to the mini focus group interviews described above, a survey was created to collect information about the characteristics and performance of Australia’s current operational BDDs. The survey had three components: (1) a validated canine personality questionnaire, (2) a questionnaire of working traits extracted from focus group discussions, and (3) ratings of working performance.

#### 4.1.1. Component One: Monash Canine Personality Questionnaire—Revised

The first section of the survey was a previously validated measure of dog personality/behavioural traits, the Monash Canine Personality Questionnaire—Revised (MCPQ-R) [22,23]. It comprises a list of descriptive words (Table 1) with a 6-point Likert response scale from “Really does not describe [Dog Name]” to “Really describes [Dog Name]”. 

#### 4.1.2. Component Two: Work Behaviour Questionnaire

The themes and constructs extracted from the focus group sessions were used to formulate questionnaire items about work-related traits that were expected to be important. Only the behaviours that all handlers could reasonably have been expected to witness were included, whereas questions about dogs’ kennel behaviours, training, and cognitive abilities were excluded. A selection of participants’ statements and examples that represented the prevalent themes were phrased as possible questionnaire statements. Based on clarity and anticipated ease of responding, statements were chosen by consensus among the experimenters and a DAWE representative. The statements were separated into overarching themes as determined in focus groups, and were then separated into the coded categories that were expected to be distinct, and condensed with a maximum of three statements from each category (presented in Table 2). These categories were then refined based on the outcomes of internal consistency testing using Cronbach’s alpha (Table 2).

For this component of the survey, participants were asked to rate their agreement with each statement on a 7-point Likert scale from “Strongly disagree” to “Strongly agree”. The end of this section included a text area for optional comments, asking, “Do you have any other comments to add?” Responses to this were not analysed, but this question was included for clarification or feedback opportunity.

#### 4.1.3. Component Three: Handler-Rated Working Performance

The final section asked handlers to rate the dog’s detection performance on a sliding scale of 1–10 with 0.1 decimal steps, with 1 representing poor and 10 representing excellent. They were asked, based on their own perceptions drawn from working with the dog, to rate the dog’s sensitivity (“How well you think they find all the targets that are present”), specificity (“How well they avoid making false responses”), and their general detection performance in the airport, mail centre, and overall. The end of this section included another text area for optional comments.

### 4.2. Survey Distribution

The survey was distributed electronically, via Qualtrics software (Qualtrics, Provo, UT), to all DAWE dog handlers. They were invited to report and rate the behaviours and performance of the individual dog(s) that they handled. The response rate was high; 88% of the currently operational dogs were reported on. A separate survey was completed for each individual dog (*n* = 36). The dogs were Labrador retrievers, 17 male and 19 female, aged 2–8 years (*M* = 67.8 months). Some handlers completed the survey for more than one dog. Because all responses were anonymous, we were unable to identify when this occurred and so made the decision to include all available data. Where there was more than one survey response for an individual dog, the responses were averaged to produce a single set of variables for that dog. This approach was taken to retain the maximum amount of information for greater accuracy. Descriptive statistics were then calculated for each new variable.

### 4.3. Data Analysis

Analyses were carried out using IBM SPSS Statistics Version 28.0.1.0 and RStudio Version 22.02.2.485 [24]. To develop a framework that captures scent detection traits relevant to BDDs, we first determined the underlying dimensions of working behaviours from the working behaviour questionnaire (Aim 1). Following this, we sought to ascertain the behavioural profile of currently operational BDDs (Aim 2). Finally, the utility of the questionnaire for investigating work-related behaviours for BDDs was assessed by its associations with the previously validated MCPQ-R (Aim 3) and whether dimensions of working behaviours were associated with performance ratings (Aim 4).

#### 4.3.1. Aim 1: Determine Underlying Dimensions of Working Behaviours

The items from the work behaviour questionnaire were analysed using both a theory-grounded method and data-driven method to investigate the underlying dimensions. First, the framework derived from focus group coding was used as the basis for the construct subscales. The internal consistencies of these constructs were calculated and, in cases where Cronbach’s α could be improved or was unacceptably low (< 0.5), were refined by separating items (Table 2). Items were averaged into a single score for each resulting category, with items reverse-coded, as marked in Table 2. Following this, an exploratory factor analysis (EFA) was conducted to determine whether the variance could be better explained with fewer dimensions. Bartlett’s test of sphericity and KMO’s test of sampling adequacy were used to determine the appropriateness of this method. An exploratory factor analysis using unweighted least squares, determined to be the most appropriate method for a small sample size, was conducted [25]. The number of factors was confirmed based on examination of the scree plot, eigenvalues > 1, and their interpretability. Orthogonal and oblique-rotated solution matrices were examined, and item loadings were found to be generally consistent between methods. An oblique rotation, Promax, was chosen due to the likelihood of correlations between factors. For each factor, a composite score was calculated from the mean of the items that had their primary loading on that factor.

#### 4.3.2. Aim 2: Determine General Behavioural Profile of Operational Dogs

Five personality variables were calculated from the MCPQ-R items as in Ley et al. [23] (see Table 1). Cronbach’s alpha was computed for each of these construct subscales to determine their reliability in this participant population. The novel work behaviour variables revealed from the EFA were calculated and assessed in the same way.

Descriptive statistics were calculated and plotted for each personality and work behaviour variable.

#### 4.3.3. Aim 3: Assess Expected Associations between Work Behaviour Questionnaire and MCPQ-R

Associations that were theoretically expected between personality variables and working behaviour variables were tested as a measure of criterion validity of the work behaviour questionnaire. Many of the variables did not have normal distributions, and therefore, Spearman’s rank correlation coefficients were used to analyse associations between them. The family-wise error rate was controlled for using Holm’s sequential procedure. 

#### 4.3.4. Aim 4: Identify Dimensions Associated with Detection Performance

The personality and work behaviour dimensions were tested for associations with detection performance using the same method as in Aim 3. Additionally, groups were determined from the dogs’ overall performance rating scores, allocated into three equal groups of the lowest, middle, and highest ratings. These groups were used to illustrate trends between performance and other dimensions.

### 4.4. Ethics Statement

The survey and methods were approved by the University of New England Human Ethics Research Committee (approval number HE22–018).

## 5. Survey Results

### 5.1. Aim 1: Determine Underlying Dimensions of Working Behaviours

Five of the initial theory-based constructs in the work behaviour questionnaire had acceptable (>0.7) or good (>0.8) internal consistencies. These were food motivation, play motivation, persistence, thoroughness, and environmental confidence. Others were refined into separate constructs, and are presented in Table 2. This process yielded 16 constructs in total.

A Bartlett’s test of sphericity was significant for the work behaviour items (Chi-square = 945.98, df = 406, *p* < 0.001). A Kaiser–Meyer–Olkin measure of sampling adequacy was poor, at 0.592, likely owing to the small population size, and suggesting that results from the exploratory factor analysis (EFA) should be tentatively regarded. The EFA yielded seven factors (Table 3), accounting for 73% of the variance (Table 4).

The dimensions yielded from factor analysis were meaningful, consistent with the original theoretical framework, had high internal consistencies, and were able to describe variation with fewer variables than the original constructs. As such, these factors were scored as new variables using an average composite score and used for the following analyses. Items loaded to factor six were reverse-scored and the factor labelled “search independence” for consistency and clarity.

### 5.2. Aim 2: Determine General Behavioural Profile of Operational Dogs

The descriptive statistics of each survey section are provided here as a baseline of the overall profile of the performance, behaviours, and personalities of this cohort of operational dogs. 

#### 5.2.1. Performance Ratings

**Figure 3:**
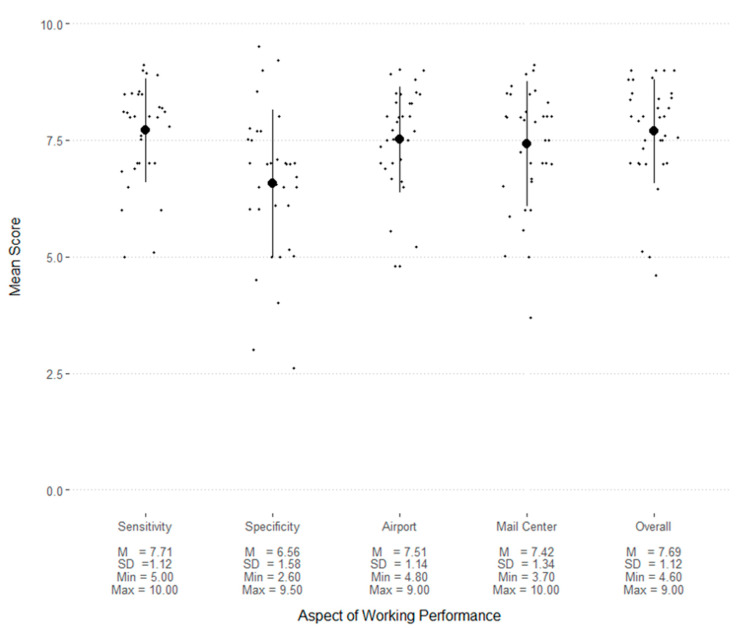
Working performance scores, where 0 indicates poor and 10 indicates excellent. The circles indicate the mean and lines indicate the standard deviation. Each point is an individual dog’s score. “Sensitivity” is the label for the item, “How well you think they find every target that is present?” “Specificity” is the label for the item, “How well they avoid making false responses?” “Airport” and “Mail Center” are the labels for items asking how well dogs perform at each of these locations. “Overall” is the label for the item asking for a rating of the dog’s overall performance.

Overall, dogs were rated highly (M ≥ 7.5) for sensitivity, performance in the airport and mail centre, and their performance overall (Figure 3).

#### 5.2.2. Work Behaviours

**Figure 4:**
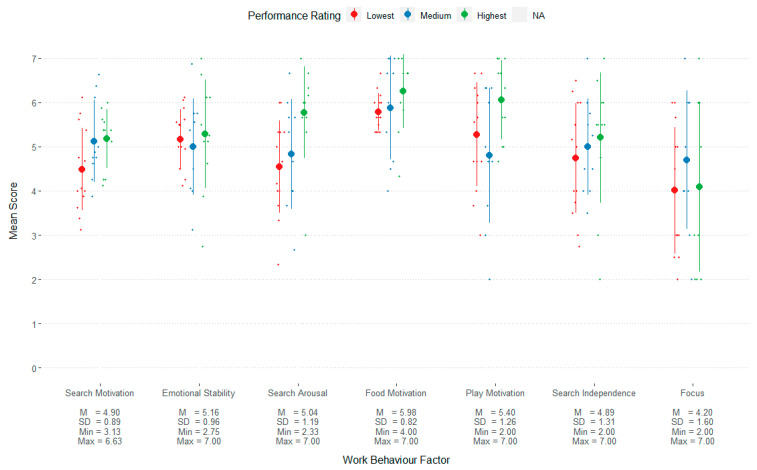
Scores of work behaviour dimensions for each group of performance rating. The circles indicate the mean and lines indicate the standard deviation. Each point is an individual dog’s score. For illustrative purposes, performance ratings groups were determined from the dogs’ overall performance rating scores allocated into three equal groups of the lowest, middle, and highest ratings. Descriptive statistics are presented below each factor label.

Handlers tended to agree that the dogs exhibited behaviours related to search motivation, emotional stability, search arousal, food motivation, play motivation, search independence, and focus, with mean ratings of >4 on these variables (Figure 4). 

#### 5.2.3. MCPQ-R

**Figure 5:**
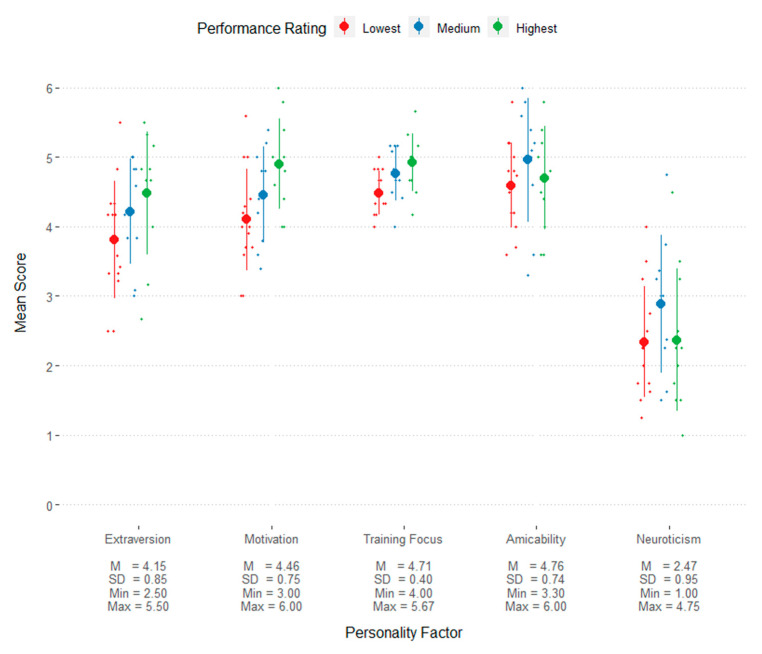
Scores of MCPQ-R personality factors for each group of performance rating. The circles indicate the mean and lines indicate the standard deviation. Each point is an individual dog’s score. For illustrative purposes, performance ratings groups were determined from the dogs’ overall performance rating scores allocated into three equal groups of the lowest, middle, and highest ratings.

Most of the MCPQ-R subscales had satisfactory internal consistencies. The calculated Cronbach alphas were moderate to high: 0.87 for extraversion, 0.78 for amicability, 0.81 for motivation, and 0.69 for neuroticism. However, the items contributing to training focus yielded poor internal consistency in this sample, with an alpha of 0.48. As such, results related to this variable should be considered with caution. The scores for each of these subscales are presented in Figure 5.

### 5.3. Aim 3: Assess Expected Associations between Work Behaviour Questionnaire and MCPQ-R

To assess the criterion validity of the work behaviour questionnaire, hypothesis testing was carried out to determine whether associations existed between this questionnaire and personality traits measured with the MCPQ-R. Reported here are tests of the expected associations based on theoretical relatedness. Most of the expected correlations were significant, and of those that were, all were in the expected direction (Table 5). All other correlation values can be found in Table S1.

### 5.4. Aim 4: Identify Dimensions Associated with Detection Performance

#### 5.4.1. Personality

Trait motivation, alternatively labelled self-assurance in the original study [22], was associated with detection performance outcomes (Table 6). Training focus, despite having low internal consistency in the current population, was also associated with overall detection performance.

#### 5.4.2. Work Behaviour

Only two of the work behaviour factors, search motivation and search arousal, were associated with ratings of overall performance (Table 7).

## 6. Discussion

The current study achieved its aim of identifying traits that are considered important for Australia’s biosecurity detector dogs (BDDs) and evaluating how these traits are expressed in the current population of operational dogs. First, a collection of traits that are relevant to the performance and welfare of BDDs was determined from focus group interviews. This process sought a more nuanced understanding of specific working behaviours and their meaning in the context of this agency. 

Subsequently, a questionnaire, designed to quantitatively assess working traits relevant for dogs in this agency, revealed several underlying dimensions of work behaviours. These dimensions were labelled search motivation, emotional stability, search arousal, food motivation, play motivation, search independence, and focus. Using these labels, we investigated the trait expressions of currently operational scent detection dogs to provide a reference for the typical behaviours of BDDs.

This methodical approach to consulting with key stakeholders will inform an ongoing project. It bolsters content validity for a new selection testing procedure and a metric by which to collect information about their day-to-day working behaviour and performance.

### 6.1. Work Behaviour Framework

A series of behaviours and descriptions were categorised into a smaller number of variables, reflecting the dimensions that underlie these work-related behaviours [26]. Initially, interview transcripts were coded and behaviours were categorised into ostensible constructs. Subsequently, a data-driven analysis of questionnaire results revealed factors that largely aligned with the original constructs but were more parsimonious, grouping the behaviours into broader but meaningful dimensions. A framework using these dimension labels was adopted for analyses and ongoing research within this population of dogs. 

The dimension of “search motivation” incorporated the largest number of questionnaire items. The items contributing to this dimension described the dogs’ eagerness, engagement, and thoroughness while searching for a target. Meanwhile, a separate factor also emerged, labelled ‘search arousal’, which described dogs’ energy and pace while working. According to focus group findings, search arousal may relate to one aspect of search motivation but may not necessarily reflect search effort. On the other hand, the behaviours clustered in the dimension of search motivation suggest effort and engagement in the task of searching. Both search motivation and search arousal reflect key descriptions of working behaviour that many focus group participants and industry professionals tend to describe as “drive” or “hunt drive” [2,7,11,27].

Behaviours indicative of “emotional stability” accounted for approximately 12% of the variation among dogs in the current population. They related to the dogs’ environmental sensitivity, stress coping, and “off-switch” behaviours. In addition to the questionnaire items intended to measure this construct, two other items, one rating dogs’ perceived calmness while searching and the other rating their ability to work with any handler, also loaded onto this factor. Connections to this dimension are logical in that calm search behaviour suggests low apparent stress, whereas especially sensitive or reactive dogs may require particular handling or the support of a known handler. Emotional stability, or aspects of it, have been investigated in other canine scent-detection research, and behaviours relating to this dimension have been considered an important indicator of dogs’ overall success in the working environment [2,11,14].

Dogs’ desire for primary reinforcers was reflected by two factors, labelled “food motivation” and “play motivation”. These constructs may underpin dogs’ initial trainability [8] and the strength of their conditioned motivation to search. 

“Search independence” emerged as a separate factor and reflected dogs’ tendency not to rely on handler input while working. According to focus group interviews, this working trait likely relates to a dog’s self-assurance and confidence to make decisions. This may relate to trait “boldness”, which has been investigated in previous working dog research and has been found to positively predict performance [28]. However, it is also possible that search independence behaviours are influenced by the dog’s training and experience in the role.

Finally, one item—“ignores distractions while searching”—loaded separately to other items. This may be because dogs’ distractibility is moderated by more than one underlying dimension, such as motivation and emotional stability, or it may be a separate dimension that no other items measured. The inclusion of this dimension requires further consideration in a future iteration of the questionnaire, as it would require more relevant items for it to be a reliable measure of this trait.

### 6.2. Traits of Operational Dogs

All of the individuals in this study are operational dogs that have completed training, and so all are considered examples of successful scent detection dogs. Indeed, when rated for overall detection performance, their mean score was above 7.5 on a scale of 10. Accordingly, we expected that trends in this population as a whole would be useful to inform our understanding of the characteristics generally required to perform the role. 

Additionally, although this narrow range of variability limits our ability to detect all associations between performance and individual traits, there was some meaningful variation in performance ratings among the dogs. As such, the observed associations and trends between behavioural characteristics and working performance were also explored. This process sought to investigate the behaviours that may predict performance potential and work towards identifying and selecting the highest performing dogs.

#### 6.2.1. Work Behaviour Traits

Overall, the average ratings of operational dogs for work-related behavioural traits were predominantly as expected (Figure 4). This cohort of dogs was rated highly for “search motivation”, “emotional stability”, and “food motivation”, with mean scores between 4.9 and 5.9 on a scale of 7, and little variation, with standard deviations below 1. This suggests that these are key traits that operational BDDs consistently demonstrate. On the other hand, more inter-individual variation was observed for “search arousal”, “play motivation”, “search independence”, and “focus”, with standard deviations between 1.2 and 1.6. This may suggest that these traits are not essential indicators of a dog’s ability to complete training and become operational, although they may still contribute to performance outcomes. Comparing successful working dogs against unsuccessful dogs will be helpful in future to determine to what extent variability in specific traits can be tolerated.

Search motivation and search arousal were both significantly positively associated with ratings of overall detection performance. It was expected that more effort and eagerness while searching would translate to better performance outcomes. Similarly, it is feasible that search arousal, which, to some extent, can reflect a dog’s enthusiasm to perform the task, would predict detection performance. However, according to interview discussions, it is likely that this relationship is not always linear and may instead have an inverted U-shape trend. While some degree of search arousal is desirable, excessive arousal could interfere with the dogs’ ability to search effectively, due to its effect on cognitive and attentional factors [6,8,29]. In this population of successful operational dogs, search arousal likely manifests at an appropriate and adaptive level, and therefore, inappropriately high levels of arousal may not be present in this sample. Overall, our findings suggest that search motivation and arousal contribute positively to perceived detection performance in an operational context. It is possible that other work-related traits may contribute to performance outcomes, but there was insufficient variability within this relatively small population to observe a statistical association. 

Emotional stability was frequently mentioned in focus group interviews but did not appear to predict detection outcomes in this population. It was hypothesised that dogs low in emotional stability would perform more poorly in a detection role than dogs high in emotional stability. Stress responses, such as fear and inability to rest, have been found to affect cognitive processes [6,30] which are believed to contribute to detection ability [10]. It is possible that the current population did not include any dogs with emotional stability so low as to compromise their performance. In the focal agency, dogs are required to work in public and potentially stressful locations, be kennelled at central facilities, and be handled by different handlers, all of which may require above-average resilience [6,31]. Therefore, there was likely a minimum threshold of emotional stability to be included in this sample, and this sampling bias may have obscured an association. Although in this specific population we did not observe an association between emotional stability and performance ratings, this dimension should not be discounted due to its likely impact on dogs’ welfare, safety, and ease of handling. 

#### 6.2.2. Personality

Operational dogs tended to be rated highly in amicability, extraversion, and motivation (alternatively labelled as self-assuredness) [22], whereas they were rated fairly low in neuroticism, as measured by the MCPQ-R (Figure 5). Trait motivation (or self-assuredness) was positively associated with performance ratings. This aligns with the perspectives of focus group participants of the descriptions of dogs suited for this role, particularly that they are non-aggressive, energetic, confident, and have high emotional stability, all of which are theoretically related to the above personality traits.

Although dogs had high scores for training focus and this trait was associated with performance outcomes, this construct had low internal consistency in this population, and so may not have offered an accurate representation of the construct. This is possibly due to interpretations of the adjectives (e.g., intelligent, obedient, reliable) by professional participants that diverge from common expectations. This highlights one of the key difficulties of identifying and measuring traits using subjective measures and reinforces the importance of considering different populations’ understandings of adjectives depending on context when using such measures. 

### 6.3. Work Behaviour Questionnaire Utility

Survey data can have the advantage of providing information based on an extended period of observation that is not always accessible or feasible to collect using objective measures [26]. This information can therefore offer a more granular measure of ongoing detection performance and variation than can be gleaned from measures such as pass-fail training outcomes, artificial detection tasks, or overall detection statistics. These objective measures are certainly an important component in validating new testing procedures; however, they can be limited in their information and in some cases can be misleading. For example, artificial tasks rarely present all of the same challenges as faced in deployment, and operational detection statistics can be impacted by differences in opportunity to make detections. As such, survey data can be a useful additional tool for the validation of behavioural tests, particularly to develop in-house assessment methods suited for a particular application or context.

We therefore assessed the utility of this work behaviour questionnaire to measure accurately the working traits in scent detection dogs and to warrant its ongoing use in this context. The face validity of the work behaviour questionnaire was supported by its reliance upon statements used by various focus group participants to describe each construct. We further assessed the validity of this questionnaire by its associations with other measures. 

The majority (6/7) of hypothesised correlations between work behaviour factors and personality factors were significant and in the expected direction (Table 5). For example, emotional stability in the workplace was related to trait neuroticism and amicability, as described by the MCPQ-R. Trait motivation (alternatively labelled as self-assurance) was positively associated with search motivation, search arousal, and search independence. Extraversion, which in the MCPQ-R suggests high energy, predicted search arousal, although not play motivation. Overall, these associations provide some evidence that the questionnaire items reflected the intended domains.

A key aim of the work behaviour questionnaire was to probe dogs’ detection behaviours and performance on a granular level. As intended, the questionnaire appeared to glean information about specific dimensions of work behaviour that are relevant to performance. The survey revealed two domains positively associated with performance ratings in this population of dogs which align with two different descriptions of “drive” cited in focus group discussions. “Drive” was the most commonly cited trait of successful scent detection dogs in the focus group discussions, and the questionnaire findings support the importance of this over-arching trait. 

A recognised limitation of the current study design is that detection performance could not be measured objectively in such a way that reliably reflected BDD general performance. As such, we relied on subjective perceptions of performance, which may not be entirely accurate or may capture only one part of the picture. However, handlers in this agency handle a variety of different dogs and so likely would be well-versed in assessing dog behaviour. Furthermore, they do not own the dogs they handle and do not carry out the initial training for the dogs, which removes much of the motivation to purposefully under- or over-estimate the dogs’ performance. Nevertheless, since unconscious biases can persist in any subjective measure, future comparisons with an objective measure of performance and work behaviour may provide a valuable indication of convergent validity.

Furthermore, the work behaviour questionnaire did not collect information about other aspects that were identified as important during focus group discussions, including their kennel behaviour, training, cognition, and physical capability. For future selection processes, these aspects will be considered and measured using other methods.

### 6.4. Future Directions

These findings will inform the development of an in-house selection testing procedure that addresses the needs of this detection role while also considering the current scientific knowledge base. This first step sought to consult with stakeholders about their experiences with BDDs, and thus is limited to a group of detection dogs that are performing the role successfully with a generally high standard of performance. As such, only general associations with performance were investigated in this instance. The findings warrant further scrutinization and predictive modelling applied to a larger group of candidate dogs with greater variation in their working suitability. This will be achieved by administering to a subsequent cohort of candidate dogs a behaviour testing procedure designed to measure relevant traits, and then comparing those behaviours to training outcomes and survey ratings of working behaviour for those dogs which become operational.

## 7. Conclusions

Our findings suggest that this methodology can identify and assess the important characteristics of a specific working dog population and role. As each working dog role is different, it is logical that different trait frameworks and behavioural examples will apply depending on the context. Consultations with stakeholders and assessment of experienced dogs in a particular role are valuable contributions to the design and advancement of behavioural testing and selection procedures.

This research revealed a collection of work-related attributes in a population of scent detection dogs used for biosecurity. These were food motivation, play motivation, search motivation, search arousal, emotional stability, search independence, and search focus. In particular, search motivation and search arousal were positively associated with detection performance ratings. These domains mirror two different examples of “drive”, as described by industry professionals in the focus group interviews. Emotional stability was another broad dimension that encompassed many important behavioural traits. Although this construct was not directly associated with detection performance, focus group discussions strongly emphasised its importance for positive welfare and handling of the dogs. Overall, there was concordance between the qualitative and quantitative methods to describe the important domains of working detector dog behaviour. 

In addition, the current questionnaire, developed to assess these work behaviour factors among detector dogs, had preliminary validity, as evidenced by correlations with validated measures of personality and associations with detection performance ratings. As such, it may be a useful tool to assess the predictive validity of other indirect measures, such as standardised behavioural testing. The ultimate aim of this will be to predict the future working behaviours and performance of unfamiliar candidate dogs based on a behavioural measure taken at a single time point. 

Measuring and understanding the individual characteristics of dogs and the association of these traits with working behaviours will pave the way to advance the selection, training, and handling of scent detection dogs. Improvements in these processes could reduce the economic and time investments of purchasing and training dogs and enhance detection performance in operational contexts. Furthermore, we could expect improved welfare outcomes by selecting dogs that are capable and motivated to perform scent detection work, resulting in enriching experiences for both the dogs and their handlers.

## Figures and Tables

**Table 1:** Descriptive words used in the MCPQ-R and the label of their underlying dimension. Scores for the items under each label were averaged to produce an overall score.

Extraversion	Motivation ^1^	Training Focus	Amicability	Neuroticism
Active	Assertive	Attentive	Easy-going	Fearful
Energetic	Determined	Biddable	Friendly	Nervous
Excitable	Independent	Intelligent	Non-aggressive	Submissive
Hyperactive	Persevering	Obedient	Relaxed	Timid
Lively	Tenacious	Reliable	Sociable	
Restless		Trainable		

^1^ Alternatively labelled as self-assurance.

**Table 2:** Statements included for each construct in the work behaviour questionnaire with the overarching themes, coded categories, and subsequently refined categories. [R] indicates the items that were reverse-scored.

Theme	Original Category	Refined Category	Statement	Internal Consistency ^1^
Motivation	Reward Motivation	Food Motivation	[Dog Name] has a strong desire for food rewards	0.845
			[Dog Name] will work really hard for a food reward	
			[Dog Name] loses interest in a food reward after a few repetitions [R]	
		Play Motivation	[Dog Name] has a strong desire for play rewards	0.852
			[Dog Name] will work really hard for a toy reward	
			[Dog Name] loses interest in a toy reward after a few repetitions [R]	
	Search Motivation	Search Motivation	[Dog Name] is always eager to start searching	0.731
			[Dog Name] will not start searching of their own accord [R]	
		Desire to sniff	[Dog Name] always tends to sniff and investigate their surroundings	-
	Search Arousal	Speed and Intensity	[Dog Name] works at a fast pace	0.882
			[Dog Name] looks highly stimulated while searching	
		Calmness	[Dog Name] appears calm while searching	-
	Focus	Engagement	[Dog Name] sometimes goes through the motions without actively searching [R]	-
		Distractibility	[Dog Name] ignores distractions while searching	-
	Persistence	Persistence	[Dog Name] wants to keep searching, even when the task is finished	0.814
			[Dog Name] sometimes gives up while searching [R]	
			[Dog Name]’s search behaviour is consistent and durable	
	Thoroughness	Thoroughness	[Dog Name] is methodical in their search pattern	0.721
			[Dog Name] searches areas superficially [R]	
			[Dog Name] searches items thoroughly	
Independence	Search Independence	Handler Consistency	[Dog Name] would work reliably for any handler	-
		Search independence	[Dog Name] relies heavily on direction from their handler [R]	0.863
			[Dog Name] often looks to their handler before indicating on an item [R]	
Emotional Stability	Environmental Confidence	Environmental Confidence	[Dog Name] works well regardless of their surroundings	0.802
			[Dog Name] is sensitive to change in their environment [R]	
			[Dog Name] recovers quickly after a stressful event	
	Off-duty coping	Off-switch	[Dog Name] can “switch off” when not working	-
		Energy use	[Dog Name] tires themselves out while off-duty [R]	-
		Crate behaviour	[Dog Name] settles calmly in their crate	-

^1^ Cronbach’s alpha.

**Table 3:** Factor loadings for each work behaviour questionnaire item. Loadings of <0.4 are not reported in this table.

Item	1	2	3	4	5	6	7
has a strong desire for food rewards				0.852			
will work really hard for a food reward				0.857			
loses interest in a food reward after a few repetitions				−0.772			
has a strong desire for play rewards					0.763		
will work really hard for a toy reward					0.838		
loses interest in a toy reward after a few repetitions					−0.912		
is always eager to start searching	0.566						
will not start searching of their own accord	−0.756						
always tends to sniff and investigate their surroundings	0.401						
works at a fast pace			1.015				
appears calm while searching		0.714					
looks highly stimulated while searching			0.973				
sometimes goes through the motions without actively searching	−0.767						
ignores distractions while searching							0.933
wants to keep searching, even when the task is finished			0.470				
sometimes gives up while searching	−0.561						
search behaviour is consistent and durable							
is methodical in their search pattern	0.467						
searches areas superficially	−0.871						
searches items thoroughly	0.947						
would work reliably for any handler		0.652					
relies heavily on direction from their handler						0.689	
often looks to their handler before indicating on an item						0.923	
works well regardless of their surroundings		0.682					
is sensitive to change in their environment		−0.673					
recovers quickly after a stressful event		0.576					
can "switch off" when not working		0.540					
tires themselves out while off-duty		−0.505					
settles calmly in their crate		0.618					

**Table 4:** Factor labels and internal consistencies of items with the highest loading onto factor.

Factor	Factor Label	No. of Items	% Variance Explained	Cronbach’s Alpha
1	Search Motivation	8	27.67	0.866
2	Emotional Stability	8	11.59	0.823
3	Search Arousal	3	9.78	0.819
4	Food Motivation	3	8.81	0.845
5	Play Motivation	3	6.14	0.852
6	Search Independence	2	1.08	0.863
7	Focus	1	4.47	-

**Table 5:** Expected correlations between work behaviour factors and personality factors with calculated correlations and significance with Holm–Bonferroni correction. The Holm alpha is the alpha needed for significance to correct for family-wise error rates.

Correlation	Spearman’s *rho*	*p*-Value	Holm Alpha
Search motivation with			
Motivation (Self-assuredness)	0.526 *	< 0.001 *	0.007
Emotional stability with			
Neuroticism	−0.617 *	< 0.001 *	0.008
Amicability	0.537 *	< 0.001 *	0.01
Search arousal with			
Extraversion	0.444 *	0.007 *	0.025
Motivation	0.695 *	<0.001 *	0.013
Play motivation with			
Extraversion	0.111	0.519	0.05
Search independence with			
Motivation (Self-assuredness)	0.549 *	<0.001 *	0.017

* denotes significance following Holm–Bonferroni correction.

**Table 6:** Correlations between overall working performance score and work behaviour factors with calculated correlations and significance with Holm–Bonferroni correction.

Factor	Spearman’s *rho*	*p*-Value	Holm Alpha
Extraversion	0.204	0.239	0.017
Motivation	0.473 *	0.004 *	0.0125
Training Focus	0.528 *	0.001 *	0.01
Amicability	0.125	0.475	0.025
Neuroticism	−0.053	0.762	0.05

* denotes significance following Holm–Bonferroni correction.

**Table 7:** Correlations between overall working performance score and work behaviour factors with calculated correlations and significance after Holm–Bonferroni correction.

Factor	Spearman’s *rho*	*p*-Value	Holm Alpha
Search motivation	0.458 *	0.006	0.008
Emotional stability	0.218	0.209	0.025
Search arousal	0.491 *	0.003	0.007
Food motivation	0.317	0.064	0.01
Play motivation	0.230	0.184	0.0167
Search independence	0.256	0.138	0.0125
Focus	0.173	0.319	0.05

* denotes significance following Holm–Bonferroni correction.

## Data Availability

The data presented in this study are available on request from the corresponding author. The data are not publicly available due to confidentiality requirements.

## Supplementary Materials

The following supporting information can be downloaded at: https://www.mdpi.com/article/10.3390/ani13030504/s1. Table S1. A correlation table depicting Spearman’s rho correlation coefficients and significance values between MCPQ-R variables and work behaviour questionnaire variables. Highlighted are the expected correlations based on theoretical relatedness.
