# Supplementary material for: Defining the Characteristics of Successful Biosecurity Scent Detection Dogs
Source: Animals (Basel). 2023 Jan 31;13(3):504. doi: 10.3390/ani13030504 (PMC9913823; doi:10.3390/ani13030504)
Supplement: Supplementary file 1 [file animals-13-00504-s001.zip › animals-2128378-supplementary.pdf]

Table S1. A correlation table depicting Spearman's rho correlation coefficients and significance values between MCPQ-R variables and work behaviour questionnaire variables. Highlighted are the expected correlations based on theoretical relatedness.

		Search motivation	Emotional stability	Search arousal	Food motivation	Play motivation	Search independence	Focus
Extraversion	Rho	.106	-.069	.444	.013	.111	.006	-.105
	Sig.	.537	.688	.007	.941	.519	.973	.540
Motivation	Rho	.526	.509	.695	.224	.270	.549	.373
	Sig.	<.001	.002	<.001	.189	.112	<.001	.025
Trainability	Rho	.506	.422	.462	.061	.307	.402	.353
	Sig.	.002	.010	.005	.723	.069	.015	.035
Amicability	Rho	.329	.537	.218	.144	.075	.177	.262
	Sig.	.050	<.001	.202	.403	.666	.302	.122
Neuroticism	Rho	-.473	-.617	-.293	-.169	-.279	-.389	-.162
	Sig.	.004	<.001	.083	.324	.099	.019	.346
